# Absence of association between early antibiotic exposure and short-term adverse outcomes in very preterm infants: a single-center retrospective study

**DOI:** 10.3389/fped.2025.1563979

**Published:** 2025-03-17

**Authors:** Laura Fillistorf, Giorgia Carra, Raphaël Matusiak, Varvara Dimopoulou, Jérémie Despraz, Sylvain Meylan, Eric Giannoni

**Affiliations:** ^1^ Clinic of Neonatology, Department Woman-Mother-Child, Lausanne University Hospital and University of Lausanne, Lausanne, Switzerland; ^2^ Biomedical Data Science Center, Department of Innovation and Clinical Research, Lausanne University Hospital and University of Lausanne, Lausanne, Switzerland; ^3^ Infectious Diseases Service, Department of Medicine, Lausanne University Hospital and University of Lausanne, Lausanne, Switzerland; ^4^ Swiss Data Science Center, Swiss Federal Institute of Technology in Lausanne, Lausanne, Switzerland

**Keywords:** antimicrobial stewardship, neonatal sepsis, neonatology, antibiotic use metrics, neonatal morbidities

## Abstract

**Background:**

Antibiotics save lives but also carry significant risks, including increased antimicrobial resistance, higher healthcare costs, and disruption of the microbiome. However, the association between antibiotic exposure and short-term adverse outcomes remains uncertain. Our study aimed to evaluate whether early unnecessary antibiotic exposure in the first 7 days of life of very preterm infants is linked to short-term adverse outcomes.

**Methods:**

This retrospective study included infants born below 32 weeks of gestation and hospitalized at the University Hospital of Lausanne between January 1, 2007 and December 31, 2022. Antibiotic exposure was quantified during the first seven postnatal days by the median number of days of antibiotics. Multilinear regressions and mixed effect models analyzed the association between the number of days of antibiotics and death, late-onset sepsis, necrotizing enterocolitis, severe bronchopulmonary dysplasia, severe retinopathy of prematurity and cystic periventricular leukomalacia. The primary outcome was a composite of at least one of the listed adverse outcomes, while the secondary outcomes consisted of each adverse outcome individually. Adjusted odds ratio (aOR) and *p*-value were calculated.

**Results:**

We included 1,398 preterm infants. The median gestational age was 29 weeks (IQR: 27–30) and the median birthweight was 1,144 grams (895–1,420). The median number of days of antibiotics declined by 53%, from 4 days in 2007 to 1.9 days in 2022 (*p* < 0.0001). The number of days of antibiotics was not associated with the composite outcome [aOR: 0.97 (0.82–1.17), *p* = 0.80, adjusted *p* = 0.80] or any of the following adverse outcomes: mortality [aOR: 1.10 (0.78–1.55), *p* = 0.58, adjusted *p* = 0.69], late-onset sepsis [aOR: 0.74 (0.59–0.93), *p* = 0.01, adjusted *p* = 0.07], necrotizing enterocolitis [aOR: 1.22 (0.86–1.74), *p* = 0.26, adjusted *p* = 0.65], severe bronchopulmonary dysplasia [aOR: 1.12 (0.88–1.42), *p* = 0.36, adjusted *p* = 0.65], severe retinopathy of prematurity [aOR: 1.34 (0.65–2.78), *p* = 0.43, adjusted *p* = 0.65], and cystic periventricular leukomalacia [aOR: 1.02 (0.69–1.99), *p* = 0.91, adjusted *p* = 0.91].

**Conclusion:**

We found no association between early antibiotic exposure and short-term adverse outcomes.

## 1 Introduction

Antibiotics are the most commonly prescribed drugs in neonatology units (1). Half of hospitalized neonates receive antibiotics (2), with rates above 70% in very preterm infants (3–7). This high proportion is attributed to the non-specific clinical signs of infection, the low diagnostic accuracy of biomarkers, the vulnerability of neonates to infections, and the physicians' fear of missing a case of sepsis (8). The burden of neonatal sepsis is high, with substantial mortality and morbidity (9, 10). While prompt initiation of antibiotics can save lives, antibiotic prescription is often disproportionate (11). In fact, the majority of neonates treated for suspected sepsis do not have a proven infection (12). This overexposure to antibiotics has significant consequences. It not only contributes to antibiotic resistance—growing threat prompting urgent action by the World Health Organization (13)—but also disrupts the microbiome, potentially contributing to the development of inflammatory diseases later in life, such as gastrointestinal disorders and asthma (11, 14–16). In response to these concerns, neonatal units are implementing antimicrobial stewardship efforts to reduce antibiotic exposure (17, 18).

The association between antibiotic exposure and short-term adverse outcomes remains unclear. While numerous studies have reported associations between antibiotic exposure and death, necrotizing enterocolitis, sepsis, and bronchopulmonary dysplasia (3, 19–24), other studies did not find such associations (4, 25, 26).

Given the ongoing controversy surrounding this topic, our study aimed to rigorously evaluate a potential association between potentially unnecessary early antibiotic exposure and major short-term adverse outcomes in very preterm infants. Additionally, we examined the evolution of early antibiotic exposure over a 15-year period.

## 2 Materials and methods

### 2.1 Study population

This retrospective study included infants born before 32 weeks of gestation and hospitalized at the neonatal unit of the University Hospital of Lausanne between January 1, 2007 and December 31, 2022. Neonates whose parents or legal guardians refused general consent for research were excluded from the analysis. This study was approved by the ethics committee of Canton de Vaud (CER 2022-00528). Neonates born outside of the University Hospital of Lausanne, as well as neonates who died or were transferred to another hospital during the first 7 days of life (DOL), were excluded from this analysis, as adverse outcomes occurring after this period could not be assessed for these patients. Since our study focused on unnecessary exposure to antibiotics, neonates with a diagnosis of culture-proven sepsis within the first seven DOL or necrotizing enterocolitis (NEC) Bell stage ≥2 in the first seven DOL were also excluded. Data on demographics and antibiotic treatments were extracted from the electronic health record system.

### 2.2 Definition of adverse outcomes

The primary outcome was a combination of the following major short-term adverse outcomes occurring after 7 DOL: death, culture-proven late-onset sepsis (LOS), NEC, severe bronchopulmonary dysplasia (BPD), severe retinopathy of prematurity (ROP) and cystic periventricular leukomalacia (PVL). Culture-proven LOS was defined as bacteremia occurring after 72 h of life (9, 27). NEC was defined as Bell stage ≥2 (27, 28). BPD was defined as >28 days of oxygen and requirement for ≥30% of oxygen and/or positive pressure ventilation at 36 weeks postmenstrual age (27, 29). ROP was defined as stage 3 or more or any stage with laser treatment (27, 30). Intraventricular hemorrhage (IVH) was not included as a measured outcome since it mostly occurs within the first 7 DOL. Secondary outcomes included each of these adverse outcomes assessed individually.

### 2.3 Metrics of antibiotic exposure

Throughout the study period, amoxicillin and gentamicin were administered as empirical therapy for suspected early-onset sepsis, while vancomycin and gentamicin were used for suspected late-onset sepsis. Continuous quality improvement interventions were implemented to promote the rational use of antibiotics and minimize the prescription of broad-spectrum agents. All antibiotics administered by intravenous, enteral, intramuscular, or intraosseous routes were recorded. Antibiotic exposure in the neonatal unit was calculated with three metrics: (1) the percentage of patients receiving antibiotics at least once during the first 7 DOL; (2) the days of antibiotics (DoA) received the first postnatal week, measured as the median number of days with at least one antibiotic administration; (3) the days of therapy (DOT), measured as the number of treatment days (dependent on the number of treatments) during the first 7 DOL per 1,000 patient-days (31).

### 2.4 Statistical analysis

Baseline clinical characteristics were described using the median and the interquartile range (IQR) for continuous variables, and absolute and relative frequencies for categorical variables. We used the Mann Kendall test to assess statistical changes in antibiotic exposure and changes in adverse outcomes over the years.

We stratified preterm infants in four groups, according to DoA: no antibiotics, 1–2 days, 2–5 days, and 5–7 days. Descriptive univariate analyses were performed to compare the four groups using Chi-square test for categorical data, analysis of variance (ANOVA) for normally distributed continuous variables and Kruskal–Wallis for non-normally distributed continuous variables. We performed pairwise comparisons to assess differences between groups, with a focus on comparing those who received antibiotics with those who did not.

We optimized multilinear regression models to assess the association between DoA and adverse outcomes, first by categorizing DoA into the four predefined groups and then by treating DoA as a continuous variable. The model was adjusted for the following confounders: complete course of antenatal steroids, multiple pregnancies, delivery mode, gestational age, birthweight, gender, Apgar score at 5 min, and ventilation during the first 7 DOL. The adjusted odds ratio (aOR) for a 10% increase in DoA was calculated, along with the corresponding 95% confidence interval (CI). Additionally, mixed-effects models were performed, incorporating both fixed effects, using the same confounders as in the model, and random effects to account for variability in outcomes across different study years. A Benjamini-Hochberg correction was applied to all analyses to adjust for multiple comparisons and reduce the likelihood of falsely identifying significant differences. Analyses were performed using R Studio 2023.06.0-421.

## 3 Results

### 3.1 Demographics

**Figure 1:**
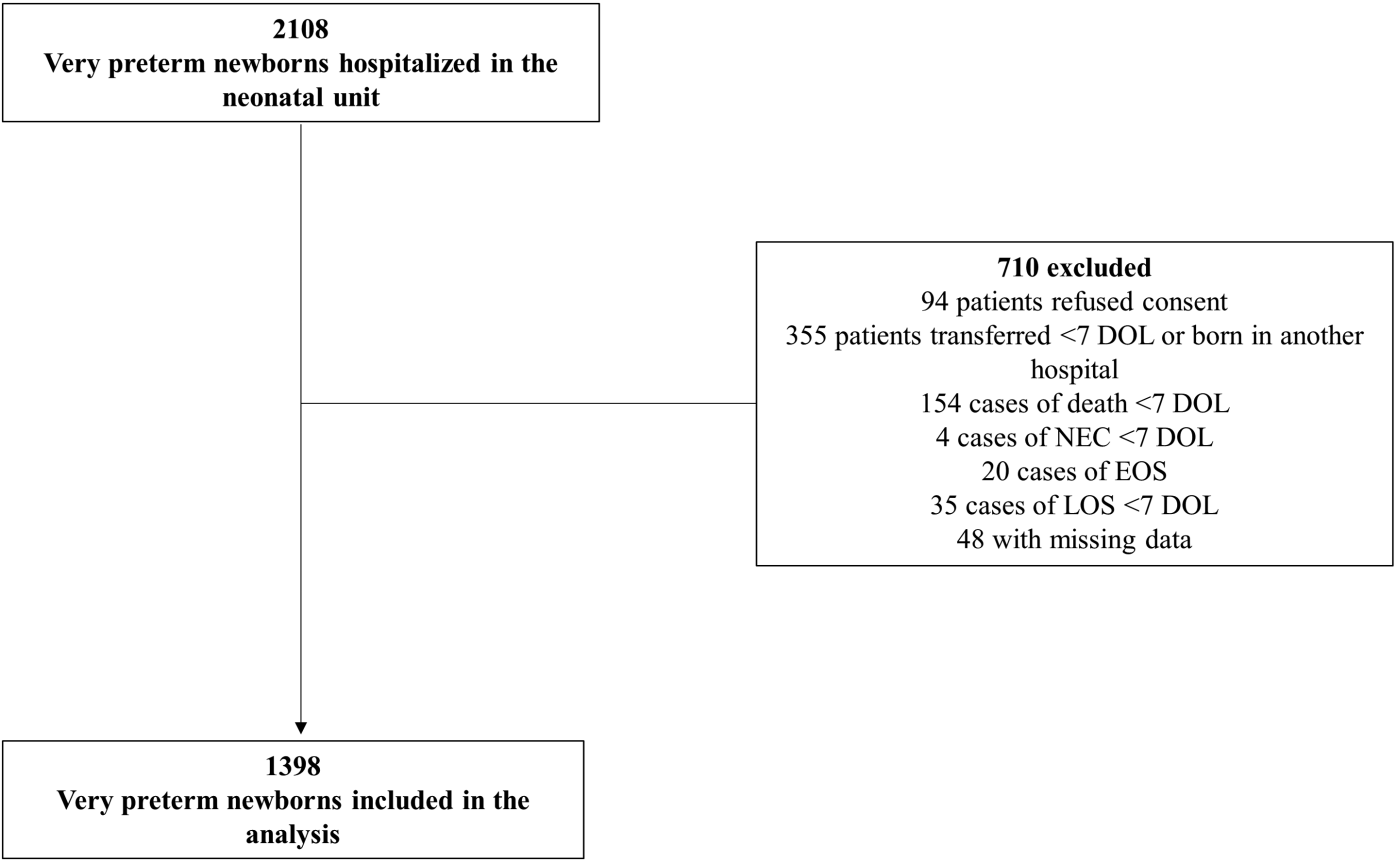
Flowchart of patient inclusion and exclusion criteria. DOL, days of life; NEC, necrotizing enterocolitis; EOS, early-onset sepsis; LOS, late-onset sepsis.

A total of 1,398 very preterm infants were included in the study (Figure 1). The median gestational age of the cohort was 29 weeks (IQR: 27–30) and the median birthweight was 1,144 grams (IQR: 895−1,420). Preterm infants with higher DoA had a lower gestational age (*p* < 0.001), a lower birthweight (*p* < 0.001) and the higher rate of mechanical ventilation within the first 7 DOL (*p* < 0.001) (Table 1).

**Table 1:** Demographic and clinical characteristics of very preterm infants according to days of antibiotics.

Characteristics	All patients	No antibiotics	DoA 1–2	DoA 2–5	DoA 5–7	*p* value^a^
Number of patients	1,398	331 (24%)	121 (9%)	637 (46%)	309 (22%)	<0.001
Antenatal steroids^b^	1,147 (82)	288 (87)	85 (70)	532 (84)	242 (78)	<0.001
Multiples	482 (34)	104 (31)	40 (33)	246 (39)	92 (30)	<0.001
Cesarean section	1,034 (74)	322 (97)	79 (65)	416 (65)	217 (70)	<0.001
Gestational age, weeks	29 (27–30)	30 (29–31)	29 (28–30)	29 (28–30)	28 (26–30)	<0.001
Birthweight, grams	1,144 (895–1,420)	1,120 (900–1,360)	1,207 (875–1,500)	1,200 (950–1,480)	1,025 (810–1,310)	<0.001
Male sex	663 (47)	182 (55)	47 (39)	305 (48)	129 (42)	<0.001
5 min Apgar score	8 (7–9)	9 (7–9)	9 (7–9)	8 (7–9)	8 (6–9)	<0.001
Ventilation^c^ <7 DOL	633 (45)	104 (31)	50 (41)	258 (40)	221 (72)	<0.001

Categorical variables are presented as frequencies (%), continuous variables as median (IQR).

DoA, days of antibiotics.

^a^ Chi-square test for categorical data, ANOVA for normally distributed continuous variables and Kruskal–Wallis for non-normally distributed continuous variables.

^b^ Complete course of antenatal steroids.

^c^ At least one day of invasive ventilation.

### 3.2 Early unnecessary antibiotic exposure

**Figure 2:**
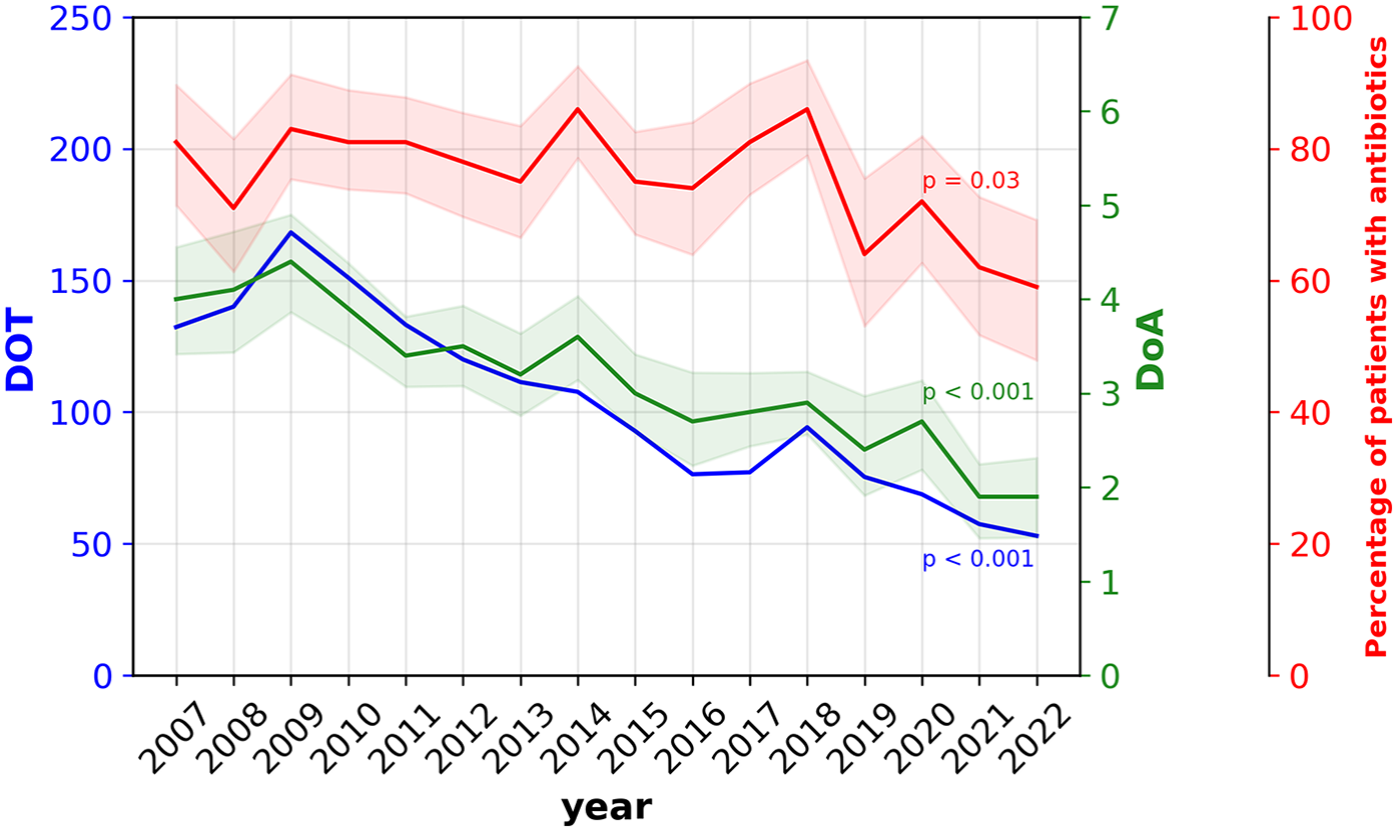
Evolution of early antibiotic exposure the first 7 postnatal days. DoA, days of antibiotics; DOT, days of therapy; Percentage of patients, percentage of very preterm infants who received at least once antibiotics during their first 7 postnatal days.

Between January 1, 2007 and December 31, 2022, DoA declined by 53% (from 4 to 1.9 days, *p* < 0.001), DOT decreased by 60% (from 132 to 53, *p* < 0.001), and the percentage of patients treated with antibiotics decreased by 28% (from 81% to 59%, *p* = 0.032) in those who survived beyond 7 DOL and did not develop EOS, LOS and/or NEC in the first week of life (Figure 2).

### 3.3 Association with adverse outcomes

The occurrence of adverse outcomes remained stable over the years (Supplementary Figure S1). In univariate analysis, the group with more than 5 days of antibiotics had a significantly higher rate of the composite outcome (*p* < 0.001), severe BPD (*p* < 0.001), NEC (*p* = 0.047) and mortality (*p* = 0.001) than the group without antibiotic exposure (Table 2). However, when correcting for confounders in a multilinear regression, there was no association between DoA groups and adverse outcomes (Supplementary Table S1).

**Table 2:** Univariate analysis of short-term outcomes in very preterm infants according to days of antibiotics.

Characteristics	All patients	No antibiotics	DoA 1–2	DoA 2–5	DoA 5–7	*p* value^a^
Number of patients	1,398	331 (24%)	121 (9%)	637 (46%)	309 (22%)	<0.001
Composite outcome	291 (21)	45 (14)	26 (21)	107 (17)	113 (37)	<0.001
Mortality >7 DOL	53 (4)	4 (1)	6 (5)	20 (3)	23 (7)	0.001
LOS >7 DOL	116 (8)	21 (6)	14 (12)	50 (8)	31 (10)	0.71
NEC >7 DOL	44 (3)	4 (1)	5 (4)	19 (3)	16 (3)	0.047
Severe BPD	135 (10)	17 (5)	11 (9)	48 (8)	59 (19)	<0.001
Severe ROP	13 (1)	0 (0)	1 (1)	5 (1)	7 (2)	0.11
Cystic PVL	32 (2)	8 (2)	1 (1)	10 (2)	13 (4)	1.00

DoA, days of antibiotics.

^a^ Pairwise comparison: Group DoA 5–7 compared to the group without antibiotics.

A multilinear regression model analyzing DoA as a continuous variable and correcting for cofounders showed no association with the primary composite outcome (*p* = 0.80) or individual major short-term adverse outcomes (Supplementary Table S2). A generalized mixed model adjusting for random effects (year of birth) indicated that DoA was not significantly associated with the composite outcome [aOR: 0.97 (0.82–1.17), *p* = 0.80, adjusted *p* = 0.80] or any short-term adverse outcomes; mortality [aOR: 1.10 (0.78–1.55), *p* = 0.58, adjusted *p* = 0.69]; LOS [aOR: 0.74 (0.59–0.93), *p* = 0.01, adjusted *p* = 0.07]; NEC [aOR: 1.22 (0.86–1.74), *p* = 0.26, adjusted *p* = 0.65]; severe BPD [aOR: 1.12 (0.88–1.42), *p* = 0.36, adjusted *p* = 0.65]; severe ROP [aOR: 1.34 (0.65–2.78), *p* = 0.43, adjusted *p* = 0.65]; cystic PVL [aOR: 1.02 (0.69–1.99), *p* = 0.91, adjusted *p* = 0.91] (Table 3).

**Table 3:** Multivariate analysis (mixed effect model) of short-term outcomes in very preterm infants according to days of antibiotics.

Adverse outcomes	Mixed effect model Adjusted OR (95% CI)	*p* value^a^
Composite outcome	0.97 (0.82–1.17)	0.80
Mortality	1.10 (0.78–1.55)	0.69
LOS	0.74 (0.59–0.93)	0.07
NEC	1.22 (0.86–1.74)	0.65
Severe BPD	1.12 (0.88–1.42)	0.65
Severe ROP	1.34 (0.65–2.78)	0.65
Cystic PVL	1.02 (0.69–1.99)	0.91

Model adjusted for antenatal steroids, multiple pregnancies, delivery mode, gestational age, birthweight, gender, Apgar score at 5 min, ventilation during the first 7 DOL and corrected for the year of birth.

DOA, days of antibiotics.

^a^ Corrected *p*-value according to Benjamini-Hochberg correction.

## 4 Discussion

In this 15-year study, potentially unnecessary antibiotic exposure during the first postnatal week was not associated with adverse short-term adverse outcomes in very preterm infants. While our descriptive analysis indicates a higher occurrence of the composite outcome, and single outcomes of mortality, NEC and BPD across DoA groups of preterm infants most exposed to antibiotics, an analysis of DoA as a continuous variable adjusting for key co-factors shows no association between antibiotic exposure and adverse outcomes.

Prior studies investigating association between antibiotic exposure and adverse outcomes have shown contradictory findings. Even among studies showing an association, there is substantial heterogeneity in the type and number of adverse outcomes associated with antibiotic exposure. Several studies found an association with mortality (3, 20, 22–24), others report associations with individual outcomes, such as BPD (21), or with multiple outcomes, such as BPD and death (22), NEC and death (20), or LOS and the composite outcome of death, LOS and NEC (19). Other studies found associations between antibiotic exposure and NEC, death, and BPD (3), BPD and cerebral lesions (24), or ROP, death and the composite outcome of death, severe ROP, BPD and anomalies on neuroimaging (23). This heterogeneity, both among studies reporting an association and those that do not, can be attributed to different inclusion and exclusion criteria, the use of different metrics of antibiotic exposure, different methods for adjustment for confounding factors, and differences between institutions in antibiotic prescription practices and incidence of adverse outcomes. For instance, the co-factors included in analyses differ between studies. There is substantial heterogeneity regarding adjustment for illness severity as some studies adjusted for CRIB or SNAPPE scores (3, 23, 24), others adjusted based on mechanical ventilation (19, 20, 22, 25, 32). This aspect is important, as multiple variables influence the risk of developing adverse outcomes in preterm infants. In previous reports, results are not always corrected for the years of inclusion (19–22, 24, 26, 32), which can lead to bias as clinical practices and standards of care change over time. Additionally, some studies analyzed the impact of antibiotic exposure, often calculated with the metric Antibiotic Use Ratio (AUR), over the entire hospital stay (23), while most focused on early exposure (3, 19–21). The decision to calculate AUR over the entire hospitalization period can be questioned, as adverse outcomes may develop prior to antibiotic exposure, thereby challenging the cause-and-effect relationship. Furthermore, some studies consider the duration of treatment, represented by different metrics such as days of treatment or AUR, whereas others focus on the timing of antibiotic initiation (e.g., whether antibiotics were started on day 1, day 3 or after day 7) (21, 24). In most studies, patients are grouped into categories of antibiotic exposure rather than treating the variable as continuous, which can introduce bias (3, 19, 20, 22, 25, 26). Another factor that may explain discrepant findings is local practices of antibiotic use, such as the types of antibiotics prescribed, the proportion of patients started on antibiotics, and the duration of treatment. Broad-spectrum antibiotics, for instance, are more commonly associated with adverse outcomes (22). Potentially relevant differences in local practices also include the implementation of LOS prevention bundles, and the use of probiotics, maternal milk and donor milk which can reduce the rates of adverse outcomes.

Antibiotic use during the first seven postnatal days has decreased over time in our neonatal unit, with rates now falling within the lower end of what is reported in the literature (3, 23). This reduction in antibiotic use could partially explain the lack of a significant association between antibiotic exposure and short-term adverse outcomes in our study.

This study has several limitations. As it was conducted at a single hospital site, the results may not be generalizable. Despite our large cohort, the number of preterm infants who developed major short-term adverse outcomes was relatively limited, which may have reduced our capacity to detect significant associations. We did not analyze the association between antibiotics and adverse outcomes according to the class of antibiotics. While we aimed to focus on the potential impact of potentially unnecessary antibiotics by excluding patients with proven LOS or NEC, we may still have included in our analysis infants with potential infections, such as meningitis, pneumonia, urinary tract infection, and culture-negative sepsis. Finally, by excluding patients affected by LOS and NEC during the first week of life, we could not evaluate the impact of very early antibiotics on the occurrence of these outcomes within the first 7 DOL.

## 5 Conclusion

When adjusting for demographics, severity of illness and years, unnecessary antibiotic exposure during the first week of life does not appear to increase the risk of major short-term adverse outcomes in very preterm infants. In addition, our results show that unnecessary antibiotic exposure can be decreased. While our findings do not support an independent association between early antibiotic use and short-term adverse outcomes of prematurity, it is crucial to use antibiotics judiciously to reduce the risk of emergence and spread of antimicrobial resistance and prevent long-term adverse outcomes associated with antibiotic overuse.

## Data Availability

Requests to access these datasets should be directed to laura.fillistorf@chuv.ch
