# Supplementary material for: Absence of association between early antibiotic exposure and short-term adverse outcomes in very preterm infants: a single-center retrospective study
Source: Front Pediatr. 2025 Mar 17;13:1563979. doi: 10.3389/fped.2025.1563979 (PMC11955704; doi:10.3389/fped.2025.1563979)

# *Supplementary Material*

**Supplementary Table 1** Multivariate analysis of short-term outcomes in very preterm infants according to groups of days of antibiotics.

| Adverse outcomes | Multilinear regression Adjusted OR (95% CI) | P-value[1] |
|---|---|---|
| Composite outcome | | |
| DoA 1-2 | 1.32 (0.67-2.57) | 0.42 |
| DoA 2-5 | 0.76 (0.47-1.22) | 0.25 |
| DoA 5-7 | 0.91 (0.53-1.57) | 0.73 |
| Mortality | | |
| DoA 1-2 | 2.70 (0.61-12.47) | 0.19 |
| DoA 2-5 | 1.61 (0.53-6.02) | 0.43 |
| DoA 5-7 | 1.58 (0.48-6.31) | 0.48 |
| LOS | | |
| DoA 1-2 | 1.40 (0.60-3.16) | 0.42 |
| DoA 2-5 | 0.79 (0.43-1-47) | 0.44 |
| DoA 5-7 | 0.44 (0.21-0.91) | 0.14 |
| NEC | | |
| DoA 1-2 | 3.64 (0.86-16.37) | 0.08 |
| DoA 2-5 | 2.29 (0.77-8.52) | 0.17 |
| DoA 5-7 | 2.62 (0.78-10.59) | 0.14 |
| Severe BPD | | |
| DoA 1-2 | 1.10 (0.42-2.75) | 0.85 |
| DoA 2-5 | 0.98 (0.51-1.94) | 0.96 |
| DoA 5-7 | 1.33 (0.65-2.78) | 0.43 |
| Cystic PVL | | |
| DoA 1-2 | 0.38 (0.02-2.35) | 0.38 |
| DoA 2-5 | 0.46 (0.16-1.31) | 0.14 |
| DoA 5-7 | 0.78 (0.23-2.40) | 0.66 |

Model adjusted for antenatal steroids, multiple pregnancies, delivery mode, gestational age, birthweight, gender, Apgar score at 5 minutes and ventilation during the first 7 DOL.
DoA: Days of antibiotic. 1. Corrected p-value according to Benjamini-Hochberg correction 2. Results for ROP could not be calculated due to the low number of cases.

**Supplementary Table 2** Multivariate analysis of short-term outcomes in very preterm infants according to days of antibiotics as a continuous variable.

| **Adverse outcomes** | **Multilinear regression Adjusted OR (95% CI)** | **P-value[1]** |
|---|---|---|
| Composite outcome | 0.98 (0.82-1.17) | 0.80 |
| Mortality | 1.14 (0.82-1.58) | 0.58 |
| LOS | 0.74 (0.59-0.93) | 0.07 |
| NEC | 1.22 (0.86-1.74) | 0.58 |
| Severe BPD | 1.08 (0.86 – 1.35) | 0.58 |
| Severe ROP | 1.39 (0.72-2.84) | 0.58 |
| Cystic PVL | 1.11 (0.75-1.64) | 0.58 |

Model adjusted for antenatal steroids, multiple pregnancies, delivery mode, gestational age, birthweight, gender, Apgar score at 5 minutes and ventilation during the first 7 DOL. 1. Corrected p-value according to Benjamini-Hochberg correction

**Supplementary Figure 1** Evolution of adverse outcomes between 2007 and 2022.

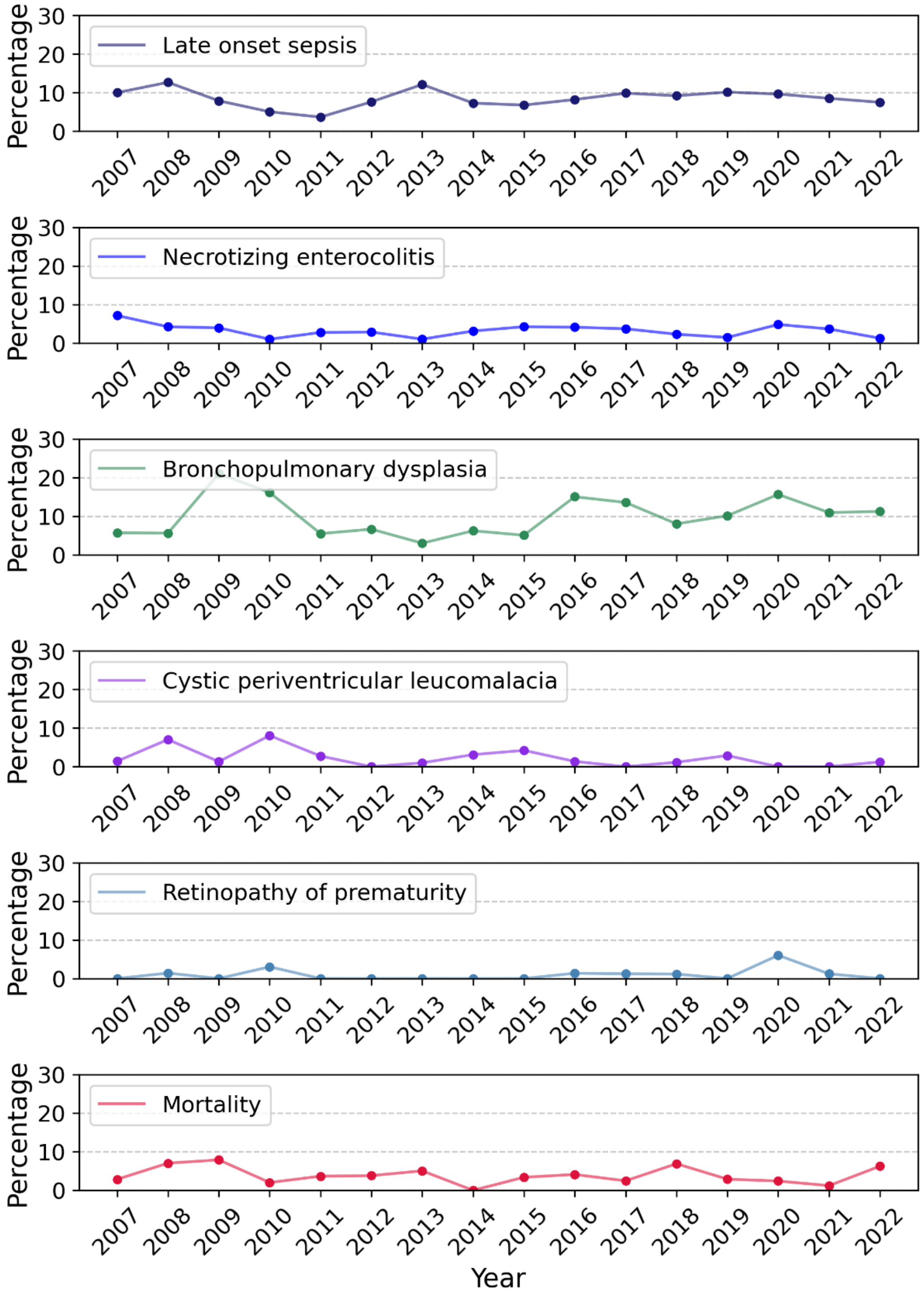

Supplement: Supplementary file 1 [file Datasheet1.pdf]
